# Effect of Peripheral Sensory Electrical Stimulation Combined With Handwriting Practice on Nondominant Handwriting Skills in Adults: An Exploratory Randomized Controlled Trial

**DOI:** 10.7759/cureus.92318

**Published:** 2025-09-14

**Authors:** Masaaki Sato, Hitoshi Mutai, Chiho Kitamori, Yuki Seike, Ayari Takano, Hiroki Sakuma, Riku Yokoyama, Jun Iwanami, Akira Sagari

**Affiliations:** 1 Division of Occupational Therapy School of Health Sciences, Shinshu University, Matsumoto, JPN; 2 Department of Rehabilitation, Ichinose Neurosurgical Hospital, Matsumoto, JPN; 3 Department of Rehabilitation, Takeshige Hospital, Nagano, JPN; 4 Department of Rehabilitation, Niiza Shiki Central General Hospital, Saitama, JPN; 5 Department of Rehabilitation, Tokyo Bay Rehabilitation Hospital, Narashino, JPN; 6 Department of Rehabilitation, Marunouchi Hospital, Matsumoto, JPN

**Keywords:** handwriting, non-dominant hand, occupational therapy, peripheral nerve stimulation, randomized controlled trial

## Abstract

Introduction

Tracing letters is recognized in occupational therapy as an effective method for improving writing skills with the non-dominant hand. Additionally, peripheral sensory nerve electrical stimulation (PES) increases corticospinal tract excitability and enhances the acquisition and retention of motor skills. This study aimed to investigate whether combining character tracing with PES can improve non-dominant handwriting ability in adults.

Methods

The participants were randomly assigned to one of three groups: handwriting with PES (PES group), handwriting only (non-PES group), and a control group. All participants were instructed to copy a sample character using a ballpoint pen with their non-dominant hand at baseline and five days after the intervention. The primary outcome was character quality assessed using computer-based character recognition software and human-rated global legibility scales. Writing speed during the copying task was the secondary outcome. The intervention groups practiced character tracing for 15 minutes per day for five consecutive days. In the PES group, stimulation was applied for 40 minutes before and 15 minutes during each handwriting session. Outcome data were tested for normality, and non-normally distributed data were log-transformed before analysis. Analysis of covariance (ANCOVA) was used to adjust for the writing speed.

Results

ANCOVA revealed a significant improvement in character quality in the PES group compared with that in the control group after the intervention. However, no significant differences were observed between the PES and non-PES groups or between the non-PES and control groups.

Conclusion

When adjusted for writing speed, handwriting practice combined with PES significantly improved non-dominant handwriting quality compared with no intervention. However, PES alone did not demonstrate a clear additional benefit over handwriting practice.

## Introduction

Writing is one of the most crucial activities of daily life. Clinical occupational therapy often addresses writing difficulties in children [1] and adults [2]. When a stroke or trauma results in the impairment of the dominant hand and functional improvement is not expected, occupational therapy is used to develop writing movements with the non-dominant hand. Our previous randomized controlled trial of healthy participants showed that tracing practice was more effective in improving writing movement ability with the non-dominant hand than upper limb functional training using a pegboard [3]. Therefore, we aimed to investigate more effective methods of tracing practice.

Recently, peripheral sensory nerve electrical stimulation (PES) [4] has found application as a form of electrical stimulation therapy to enhance motor function in rehabilitation medicine. PES enhances primary motor cortex excitability by delivering peripheral sensory input; this reduces gamma-aminobutyric acid system-mediated inhibition. This process facilitates the activation of the primary sensory cortex corresponding to the stimulated body region and the primary motor cortex [5,6]. PES, combined with motor learning tasks, improves learning effectiveness [7] and positively affects motor memory [8].

Therefore, we hypothesized that combining PES with writing practice in the dominant hand exchange may enhance the motor learning effect of the non-dominant hand, resulting in higher practice effectiveness. Our previous study [9] examined this effect on the improvement of character quality among three groups: 1) a practice method in which the PES was energized during the 20-minute tracing practice; 2) a method in which tracing practice was performed without PES energizing, and 3) a control group in which no practice was performed. However, the 20-minute PES energization time was insufficient to achieve the combined effect. A systematic review examining the effects of PES on motor learning revealed that several studies have examined the effects of PES with longer stimulus duration settings [10]. Therefore, we hypothesized that a longer stimulus duration would produce a combined effect. In the present study, we extended the stimulation time of PES and aimed to examine the combined effects of PES on writing practice in the non-dominant hand through an exploratory randomized controlled trial. This study aimed to determine whether combining PES with tracing-based handwriting practice more effectively improves non-dominant handwriting quality, specifically character quality and writing speed, compared to handwriting practice alone or no intervention in healthy adults.

## Materials and methods

We conducted an exploratory randomized controlled trial based on the Consolidated Standards of Reporting Trials (CONSORT) 2010 statement [11] to verify the beneficial effects of writing practice combined with PES on handwriting. This exploratory RCT was conducted with blinded pre- and post-evaluations. In addition, this study was conducted following the principles of the Declaration of Helsinki [12]. Ethical approval was provided by the Ethics Review Board of our institution (approval number 5516). This study was registered in the University Hospital Medical Information Network Clinical Trial Registry (UMIN000048058).

Participants

Participants were recruited via the posting of study recruitment information. In total, 36 healthy undergraduate students aged >18 years were enrolled. The exclusion criteria were defined based on our previous study [3] as follows: left-hand dominance (assessed using the Edinburgh inventory), native language other than Japanese, muscle weakness, restricted range of motion, sensory deficits in the upper limb, prior training in left-hand writing, and presence of implanted electronic devices such as pacemakers. All participants were provided with written and verbal explanations of the study, and written consent was obtained [3]. After eligibility was confirmed, participants were enrolled and randomly assigned to the PES (writing practice combined with PES, n = 12; one male), non-PES (writing practice-only, n = 12; two males), and control groups (no intervention, n = 12; two males). As this was a pilot study, a formal sample size calculation was not performed [13]. Several studies have recommended a sample size of 36 (12 participants per group) for pilot studies [14-17]. Based on these previous studies, we recruited 12 participants for each group. Participants were enrolled from October 2022 to October 2023, and follow-up assessments were concluded in November 2023.

Randomization and blinding

This study is a single-blind randomized controlled trial with evaluator blinding. Randomization was performed using a computer-created random number table [3]. Allocation was conducted by an investigator not involved in the assessment or intervention. The evaluator and intervener were blinded by assigning roles to different individuals. The character recognition rate and Global Legibility Scale (GLS) scores were evaluated after shuffling the completed papers to ensure that the evaluator was blinded to the participants’ group (PES, non-PES, and control groups) and evaluation time (pre and post) [3]. Data regarding sex and age were collected before the intervention [3]. Participants were assigned to either the PES or non-PES group without being informed which group they were assigned to, to minimize the risk of identifying the assigned intervention.

Measures

Basic participant attributes were assessed before the intervention, including age, sex, grip strength of the left hand, pinch strength (pulp pinch), and finger dexterity. To assess each participant's baseline left upper extremity function, muscle strength was evaluated using grip strength (kgf) [3] and pinch strength (kgf) [3]; finger dexterity was assessed using the O'Connor Finger Dexterity Test (Lafayette Instrument Co., Lafayette, IN) [18]. A digital grip strength meter (GT-5401; OG Wellness, Okayama, Japan) was used to determine grip strength [3]. Grip strength was measured twice, and the average value was used [3]. A pinch force meter (ST-1023; OG Wellness) was used to determine the pinch force [3]. Pulp pinch was measured twice, and the average value was used [3].

The raw score of the O'Connor Finger Dexterity Test was used to evaluate the maneuverability and dexterity of the fingers [3]. This test is a peg-placement test that has been used to evaluate the rapid manipulation of small objects [18]. It consists of a Masonite board with a molded surface comprising 100 holes (each measuring 0.47 cm in diameter) arranged in 10 rows of 10 holes (positioned 1.26 cm apart) each [18]. Three hundred and fifteen pins (length 2.54 cm; diameter 0.16 cm) lay in a well [18]. The participant was required to fill each hole with three pins as fast as possible using their dominant hand [18]. The time used for the filling of the first half of the board (five rows, 50 holes) was recorded in seconds and, thereafter, summed with the time used for the filling of the second half of the board (five rows, 50 holes) [18]. The time (seconds) taken to fill the second half of the board was multiplied by 1.1. The mean of this value and time (seconds) taken to fill the first half of the board were computed, i.e., raw score = (time for first 50 holes + (time for second half holes × 1.1))/2 [18]. The smaller the value of the raw score, the better the function [3]. Evaluations were conducted in a quiet room with a temperature of 20-25°C. A chair with a backrest and a seat height of 42 cm was used, and the table height was adjusted to one-third of the sitting height [3].

Outcome evaluations

Participants were asked to transcribe a sample character with a ballpoint pen to assess the outcomes. We used an A4-size vertical paper as the sample paper on which one character was written in one of 100 frames (1.5 × 1.5 cm) [3]. The sample included characters such as Japanese kanji, hiragana, katakana, English alphabet letters, numbers, words, affiliations, dates, and places often used daily [3]. Participants were instructed to transcribe the sample onto its corresponding frame without writing two samples in one frame [3]. In addition, the entry started when the evaluator signaled, and the time from that signal to the end of writing was measured [3]. Finally, participants were instructed to complete their writing as quickly and neatly as possible [3]. The participant’s writing posture and the position of the evaluation paper were not specified [3].

The primary outcome measures were selected based on our previous research [3], and the two indicators described below were adopted. We used the character recognition rate determined using the optical character recognition method as an objective evaluation of character shapes [3] and used the GLS as a measure of character legibility [3]. The character recognition rate [3] was calculated by first scanning the characters described by the participant into a computer, which counted the number of characters recognized using the character recognition software (Yomitorikakumei version 15; Panasonic, Osaka, Japan). Thereafter, the number of recognized characters was divided by the number of characters described. Using the GLS [3], the evaluator assessed the characters written by the participants and assigned scores of 1-5 points (good, 5 points; slightly good, 4 points; normal, 3 points; slightly poor, 2 points; poor, 1 point). The secondary outcome measure, writing speed (the number of characters written per minute), was calculated by dividing 100 characters by the time (minutes) it took to write them [3].

The pre-intervention assessment was conducted one week prior to the start of the intervention, and the post-intervention assessment was conducted after five interventions (at five days).

Interventions

The non-dominant hand was used during writing practice. These sessions lasted 15 minutes/day for five days at a time convenient for the participants [3]. The intervention schedule started on Monday and ended on Friday (five days) [3]. The rationale for setting this intervention period was based on our previous study [3], in which 15 minutes of daily writing practice was conducted for 10 days, and writing skills were significantly improved from baseline to five days. Interventions were conducted in a practice room in the facility [3]. The intervention environment was a quiet room [3]. The chair had a backrest, seat height was 42 cm, and table height was 71 cm [3]. Before the intervention started, the interveners explained the aim of the intervention and its implementation procedures to the participants [3]. The supervisor set the practice location, supervised the practice time, and observed the participants during the practice but did not interfere with the process to promote the practice [3].

The intervention group (PES and non-PES) performed the task of tracing characters. We used an A4 vertical paper with 150 frames (10 horizontals and 15 verticals), each measuring 1.5 cm × 1.5 cm; one character (26 points) was thinly printed in each frame [3]. The text content included different newspaper articles for each practice session. The characters included Japanese kanji, hiragana, katakana, English alphabet letters, and numbers. This practice was performed three times for five minutes, with a one-minute break. A ballpoint pen was used as the writing instrument [3].

**Figure 1:**
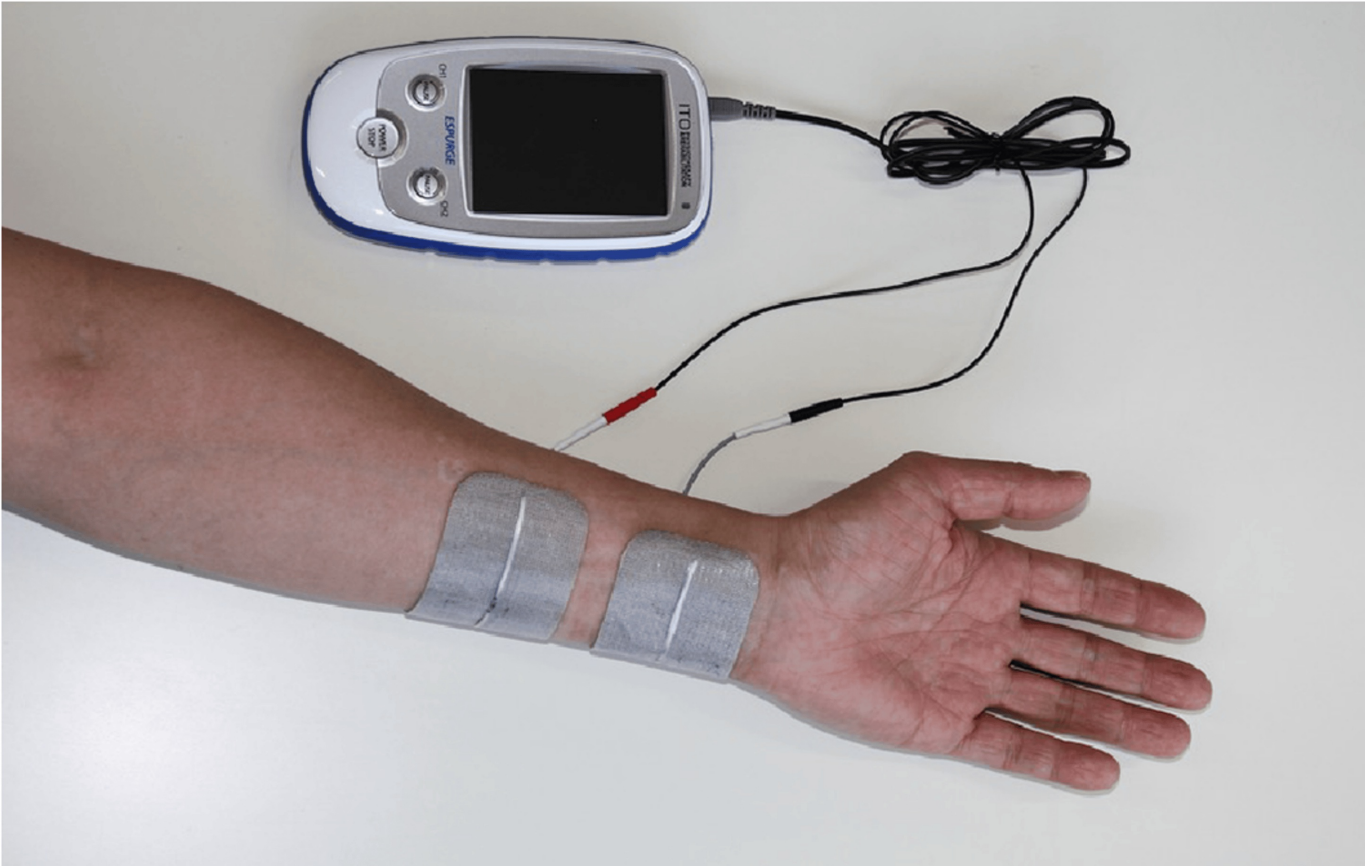
Position of electrode attachment during peripheral sensory nerve electrical stimulation

ESPURGE (ITO Physiotherapy and Rehabilitation, Co., Ltd, Kawaguchi, Japan) was used as the PES device [19]. For PES, one electrode was attached to the palmar surface, approximately two-fifths distal to the palmar wrist and elbow joint of the forearm, covering the ulnar nerves, and another electrode was attached 1 cm proximally to the first electrode (Figure 1) [19].

Although previous studies [20] have utilized diverse stimulus settings for PES, our preliminary experiments [19] prompted our use of a 10 Hz frequency and a 40-minute stimulus duration. These parameters are expected to boost primary motor cortex excitability. The electrical stimulation paradigm, excluding the frequency and stimulation time, was unified in the transcutaneous electrical nerve stimulation mode with a pulse width of 1,000 µs [19]. The stimulation amplitude varied and was adjusted to a level above the sensory threshold but below the motor threshold without causing discomfort [19]. Therefore, we assumed that the electrical pulse stimulated the sensory nerve orthodromically but not the motor nerve [19]. The actual current amplitude was 3-6 mA across the participants. In the PES group, the PES was energized for 40 minutes before the writing practice and continued during the writing practice. In the non-PES group, the electric stimulator and electrodes were attached for sham stimulation, as in the PES group, and the current was energized once but automatically terminated after two minutes.

The control group only performed the evaluation tests [3]. Moreover, the participants were instructed not to intentionally use the non-dominant hand more than they normally do in their daily life [3].

Statistical analysis

The obtained data were tested for normality using the Shapiro-Wilk test. Subsequently, appropriate statistical tests were used for analyses. Descriptive statistics (means and standard deviation (SD), medians and first-third quartiles, and frequencies and percentages) were applied based on the characteristics of each variable.

In comparing basic attributes among the three groups before the intervention, Fisher's exact probability test was used for nominal variables, and a one-way analysis of variance or the Kruskal-Wallis test was used for continuous variables, depending on the normality of the data. To examine the effects of the intervention on handwriting outcomes across groups, we conducted an analysis of covariance (ANCOVA). The primary dependent variables were the character recognition rate and character legibility scale score. As the distribution of the character legibility scale violated the assumption of normality, a log transformation was applied prior to analysis to satisfy the assumptions of ANCOVA. Given prior evidence suggesting that increased writing speed was associated with reduced handwriting quality [21], writing speed was entered as a covariate. The ANCOVA models included two between-participants factors: group (PES, non-PES, control) and time (pre-intervention, post-intervention). Additionally, there was one within-participant factor, which was also time (pre-intervention, post-intervention). Initial models tested for interaction effects among group, time, and writing speed. As none of the interactions reached statistical significance (all P > 0.05), a reduced model with only main effects and the covariate was used for the final analysis. Where significant main effects were observed (P < 0.05), Tukey’s Honestly Significant Difference (HSD) post hoc tests were performed to examine pairwise group and time differences, with correction for multiple comparisons. All statistical analyses were conducted using R (version 4.4.3; R Foundation for Statistical Computing, Vienna, Austria), and statistical significance was set at an alpha level of 0.05 (two-tailed).

## Results

The 36 participants recruited for this study were randomly assigned to the PES (n = 12), non-PES (n = 12), and control (n = 12) groups. All participants completed the pre- and post-intervention assessments, and those in the PES and non-PES groups completed the five-day intervention. No adverse events were reported.

Table 1 presents a comparison of the basic attributes of the participants at baseline. The median age of the participants was 20, 20, and 19 years in the PES, non-PES, and control groups, respectively (P = 0.080). At baseline, the median O'Connor Finger Dexterity Test scores of the non-dominant hand were 255.6, 288.0, and 267.2 for the PES, non-PES, and control groups, respectively (P = 0.789). The mean grip strength values of the non-dominant hand at baseline were 25.6 kgf (SD ± 6.3), 24.9 kgf (SD ± 5.3), and 25.1 kgf (SD ± 5.9) for the PES, non-PES, and control groups, respectively (P = 0.960). The mean pulp pinch values of the non-dominant hand at baseline were 6.9 kgf (SD ± 1.4), 7.9 kgf (SD ± 2.2), and 7.4 kgf (SD ± 2.0) for the PES, non-PES, and control groups, respectively (P = 0.453). The three groups had no significant differences regarding age, sex, finger function, grip, or pinch strength.

**Table 1:** Baseline demographics of the three groups of participants ^a^The Kruskal-Wallis test. ^b^Fisher's exact test. ^c^One-way analyses of variance (ANOVA). PES, peripheral sensory nerve electrical stimulation

Characteristic	PES group (n = 12)	Non-PES group (n = 12)	Control group (n = 12)	P-value	H-value	Chi-square	F-value
Age, years	20 (19-21)	20 (18-22)	19 (18-20)	0.080^a^	5.05	-	-
Sex, male/female	1/11	2/10	2/10	0.793^b^	-	0.465	-
Grip, kgf	25.6 ± 6.3	24.9 ± 5.3	25.1 ± 5.9	0.960^c^	-	-	0.041
Pinch, kgf	6.9 ± 1.4	7.9 ± 2.2	7.4 ± 2.0	0.453^c^	-	-	0.812
O'Connor Finger Dexterity Test scores, points	255.6 (245.1-310)	288.0 (234.1-312)	267.2 (228.8-297.3)	0.789^a^	0.474	-	-

Furthermore, 3 (group: PES, non-PES, control) × 2 (time: pre-intervention, post-intervention) ANCOVA was conducted to examine the effects of group and time on character recognition rate, with writing speed as a covariate. The results of the ANCOVA are presented in Table 2.

**Table 2:** Analysis of covariance for character recognition rate, with writing speed as a covariate PES, peripheral sensory nerve electrical stimulation

Source	Sum of squares (SS)	df	F-value	P-value
Writing speed (covariate)	4152	1	47.48	<0.001
Group (PES, non-PES, control)	729	2	4.17	0.020
Time (pre-post intervention)	3103	1	35.48	<0.001
Residuals	5860	67	-	-

The analysis revealed a statistically significant main effect for group (F(2, 67) = 4.17, P = 0.020). In addition, there was a statistically significant main effect for time, F(1, 67) = 35.48, P < 0.001, indicating an overall improvement in character recognition rate from pre-intervention to post-intervention. Writing speed was a significant covariate (F(1, 67) = 47.48, P < 0.001), confirming its association with character recognition rate. No significant interaction effects were observed among Group, Time, and writing speed (all P > 0.05). Following the significant main effects, Tukey's HSD post hoc tests were performed to examine pairwise comparisons (Table 3).

**Table 3:** Pairwise comparisons of significant main effects for character recognition rate (Tukey’s Honestly Significant Difference (HSD) post hoc test) Tukey's HSD post hoc tests were performed. PES, peripheral sensory nerve electrical stimulation

Comparison	Mean difference	95%CI (lower-upper)	P-value
A. Group sifferences			
Non-PES group vs. PES group	-4.98	-11.28 - 1.33	0.148
Control group vs. PES group	-7.84	-14.15 - 1.54	0.011
Control group vs. non-PES group	-2.87	-9.17 - 3.44	0.522
B. Time differences			
Post vs. pre	12.92	8.64 - 17.20	<0.001

Regarding the main effect of group, post hoc tests indicated that the control group had a significantly lower character recognition rate compared to the PES group (mean difference = -7.84, 95% confidence interval, CI (-14.15, -1.54), P = 0.011). No significant differences were found between the PES and non-PES groups (P = 0.148) and between the non-PES and control groups (P = 0.522). Regarding the main effect of time, character recognition rate significantly increased from pre-intervention to post-intervention across all groups (mean difference = 12.92, 95% CI (8.64, 17.20), P < 0.001).

In addition, 3 (group: PES, non-PES, control) × 2 (time: pre-intervention, post-intervention) ANCOVA was conducted to examine the effects of group and time on GLS, with writing speed as a covariate. The results of the ANCOVA are presented in Table 4.

**Table 4:** Analysis of covariance for global legibility scale, with writing speed as a covariate PES, peripheral sensory nerve electrical stimulation

Source	Sum of squares (SS)	df	F-value	P-value
Writing speed (covariate)	2.09	1	19.01	<0.001
Group (PES, non-PES, control)	1.26	2	5.70	0.005
Time (pre-post intervention)	2.41	1	21.91	<0.001
Residuals	7.37	67	-	-

The analysis revealed a statistically significant main effect for Group (F(2, 67) = 5.70, P = 0.005). Further, there was a statistically significant main effect for Time (F(1, 67) = 21.91, P < 0.001), indicating an overall improvement in GLS from pre-intervention to post-intervention. Writing speed was a significant covariate (F(1, 67) = 19.01, P < 0.001), confirming its association with GLS. No significant interaction effects were observed among group, time, and writing speed (all P > 0.05). Following the significant main effects, Tukey's HSD post hoc tests were performed to examine pairwise comparisons (Table 5).

**Table 5:** Pairwise comparisons of significant main effects for global legibility scale (Tukey’s Honestly Significant Difference (HSD) post hoc test). Tukey's HSD post hoc tests were performed. PES, peripheral sensory nerve electrical stimulation

Comparison	Mean difference	95% CI (lower-upper)	P-value
A. Group differences			
Non-PES group vs. PES group	-0.09	-0.32 - 0.14	0.609
Control group vs. PES group	-0.32	-0.55 - -0.09	0.014
Control group vs. non-PES group	-0.23	-0.45 - 0.00	0.055
B. Time differences			
Post vs. pre	0.36	0.20 - 0.52	<0.001

For the main effect of group, post hoc tests indicated that the control group had a significantly lower GLS score compared to the PES group (mean difference = -0.32, 95% CI (-0.55, -0.09), P = 0.004). No significant differences were found between the PES and non-PES groups (P = 0.609) and between the non-PES and control groups (P = 0.055). Regarding the main effect of Time, GLS score significantly increased from pre-intervention to post-intervention across all groups (mean difference = 0.36, 95% CI (0.20, 0.52), P < 0.001).

**Figure 2:**
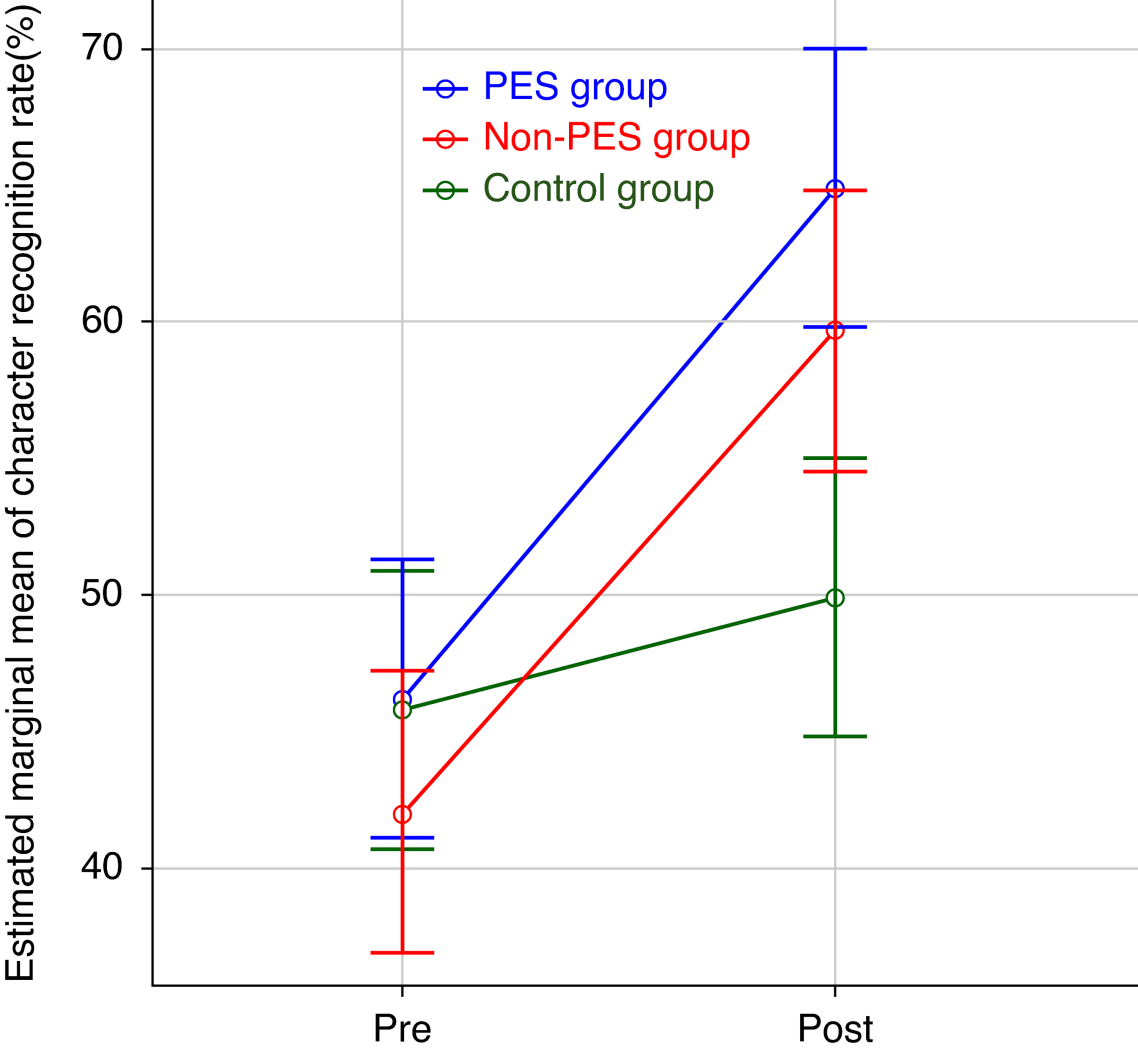
Estimated marginal means of character recognition rate by group and time Error bars represent 95% confidence intervals of the estimated marginal means. The analysis of covariance (ANCOVA) indicated significant main effects for Group (F(2, 67) = 4.17, P = 0.020) and Time (F(1, 67) = 35.48, P < 0.001). Writing speed was included as a covariate.

**Figure 3:**
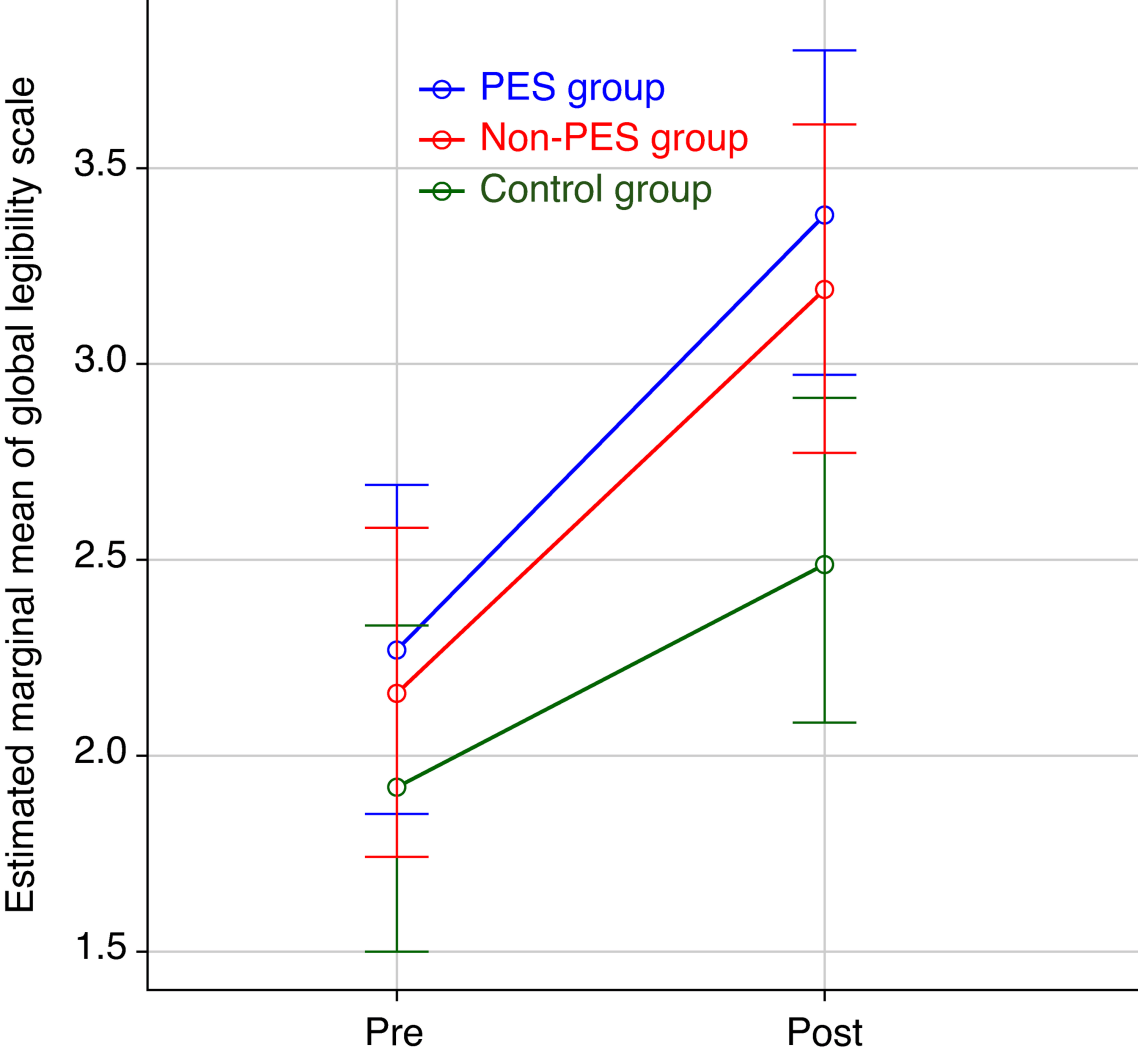
Estimated marginal means of Global Legibility Scale (GLS) by group and time Error bars represent 95% confidence intervals of the estimated marginal means. The analysis of covariance (ANCOVA) indicated significant main effects for Group (F(2, 67) = 5.70, P = 0.005) and Time (F(1, 67) = 21.91, P < 0.001). Writing speed was included as a covariate.

To visually represent the effects of group and time on the outcomes after adjusting for writing speed, the estimated marginal means for both character recognition rate and GLS score are presented in Figure 2 and Figure 3, respectively.

## Discussion

In this exploratory randomized controlled trial, we examined the effect of combining PES with tracing practice in writing with the non-dominant hand. During a five-day intervention period, the slope of change in character quality before and after the intervention was significantly greater in the PES and non-PES groups than in the control group. However, when writing speed was used as a covariate, the PES group showed a significant improvement in letter quality compared to the control group, and there were no significant differences between the PES and non-PES groups and between the non-PES and control groups. Therefore, although this study did not find a clear intervention effect of combining PES with writing practice, it suggests that combining PES with writing practice may be useful when considering writing speed.

When writing characters, writing speed, in addition to the quality of the characters, is important for improving work efficiency. Originally, it was reported that writing speed tends to reduce when the writer attempts to write well [21]. Besides, our previous study [3] found that the group that performed tracing exercises tended to show a decrease in writing speed after the intervention compared to the control group. However, when we performed ANCOVA with writing speed as a covariate, only the PES group showed a significant improvement in letter quality compared to the control group. This suggests that PES may lead to improved character quality without decreasing writing speed. McDonnell and Ridding reported that in an experiment where healthy participants performed an upper extremity functional task using the grooved pegboard test, the PES stimulation group showed significantly shorter performance times than did the control group [7,22]. The effect of PES before upper extremity functional training on improving the speed of performance of upper extremity functional tasks has been demonstrated in healthy participants and patients with chronic stroke in a double-blind crossover study [23] and in patients with subacute stroke [24]. In addition, the effect of performing PES during upper extremity functional tasks has been tested in an exploratory randomized controlled trial in patients with subacute stroke [25]. It has been suggested that the performance improvements reported in previous studies may reflect a neuroplastic mechanism involving changes in cortical motor excitability in response to afferent inputs generated by PES [26]. Therefore, the combined use of PES before and during writing practice is expected to improve character quality and reduce writing speed. These behavioral improvements may, at least in part, reflect neuroplastic adaptations facilitated by sensory stimulation. However, as the present study did not include neurophysiological measures, such interpretations remain speculative and require further validation.

Furthermore, although this study was conducted in healthy young adults, the findings may have implications for clinical populations with motor impairments, such as stroke survivors or older adults experiencing age-related decline in fine motor skills. Because writing speed and character quality are both crucial for daily communication and independence, an intervention that can enhance handwriting quality without reducing writing speed may be particularly beneficial in these groups. Previous studies have demonstrated the positive effects of PES on upper extremity motor performance in patients with stroke [23-25]. Building on these results, the present findings suggest that combining PES with handwriting practice may provide a feasible rehabilitation strategy to improve fine motor control and writing efficiency in clinical settings. Future studies should investigate the efficacy and long-term effects of this combined approach in patient populations with motor impairments to confirm its clinical utility.

Limitations and future research

This study has several limitations. First, based on the results of previous studies and the present study, there is a clear conflicting relationship between character quality and writing speed, indicating the need to re-examine the way instructions are given during evaluation tasks and task structure. Second, the intervention period (five days) was short, and the task of tracing characters varied across sessions, resulting in slight differences in difficulty. In future studies, it is necessary to establish a longer intervention period and review and standardize the intervention content. Furthermore, because this study did not examine the long-term effects of the intervention, follow-up research on the effects of the intervention is warranted. Third, further studies on implementing PES combined with writing practice are warranted. PES stimulation at a stimulus frequency of 10 Hz for 40 minutes before writing practice was a stimulus setting that increased cortical motor cortex excitability in our preliminary experiment [19]; however, we have not compared the effects of PES stimulation during writing practice with those of other stimulus conditions. The effects of PES combined with voluntary exercise may vary depending on the contraction mode of the voluntary exercise [27]. Therefore, it is important to examine the stimulus settings of PES that are suitable for writing movements in the future, as there may be differences in the effects of PES depending on how the ballpoint pen is held and moved, and peripheral joint movements. Furthermore, this study did not examine differences in primary motor cortex excitability between the PES and non-PES groups before and after the intervention; therefore, it cannot provide direct physiological evidence to explain the potential mechanisms of PES. Future studies incorporating neurophysiological measures will be necessary to substantiate the behavioral findings of this study. In addition, because this study did not examine the long-term effects of the intervention, longitudinal follow-up studies are warranted to clarify the persistence of PES-related benefits. Finally, this study included only healthy young adults; therefore, the results may not apply to clinical populations (e.g., patients with stroke) or older adults.

## Conclusions

In the present exploratory randomized controlled trial, we preliminarily examined the combined effects of PES on writing practice in the non-dominant hand. When considering writing speed, the intervention method combining the writing method and PES may improve writing quality compared to the control group. However, no clear effect was observed from combining PES and the writing method. These results indicate that although PES can contribute to the refinement of certain aspects of motor performance, its overall effect on writing tasks remains limited. The findings highlight the importance of reconsidering task design and instructional approaches, as trade-offs between writing speed and character quality were observed. Future investigations exploring optimal PES stimulus settings and their interaction with specific task characteristics will be required to clarify the practical relevance of PES for rehabilitation and skill acquisition.
